# Temporal Processing in Audition: Insights from Music

**DOI:** 10.1016/j.neuroscience.2017.10.041

**Published:** 2018-10-01

**Authors:** Vani G. Rajendran, Sundeep Teki, Jan W.H. Schnupp

**Affiliations:** a Auditory Neuroscience Group, University of Oxford, Department of Physiology, Anatomy, and Genetics, Oxford, UK; b City University of Hong Kong, Department of Biomedical Sciences, 31 To Yuen Street, Kowloon Tong, Hong Kong

**Keywords:** BI, beat induction, EEG, electroencephalography, ERPs, event-related potentials, IOI, inter-onset interval, NMA, negative mean asynchrony, SMS, sensorimotor synchronization, SSA, stimulus-specific adaptation, music psychology, sensorimotor synchronization, beat perception, rhythm perception, auditory scene analysis, temporal prediction

## Abstract

Music is a curious example of a temporally patterned acoustic stimulus, and a compelling pan-cultural phenomenon. This review strives to bring some insights from decades of music psychology and sensorimotor synchronization (SMS) literature into the mainstream auditory domain, arguing that musical rhythm perception is shaped in important ways by temporal processing mechanisms in the brain. The feature that unites these disparate disciplines is an appreciation of the central importance of timing, sequencing, and anticipation. Perception of musical rhythms relies on an ability to form temporal predictions, a general feature of temporal processing that is equally relevant to auditory scene analysis, pattern detection, and speech perception. By bringing together findings from the music and auditory literature, we hope to inspire researchers to look beyond the conventions of their respective fields and consider the cross-disciplinary implications of studying auditory temporal sequence processing.

We begin by highlighting music as an interesting sound stimulus that may provide clues to how temporal patterning in sound drives perception. Next, we review the SMS literature and discuss possible neural substrates for the perception of, and synchronization to, musical beat. We then move away from music to explore the perceptual effects of rhythmic timing in pattern detection, auditory scene analysis, and speech perception. Finally, we review the neurophysiology of general timing processes that may underlie aspects of the perception of rhythmic patterns. We conclude with a brief summary and outlook for future research.

## Highlights

• What music psychology reveals about the natural bounds of human temporal processing.
• Psychoacoustics of beat perception.
• Neurophysiology of beat perception.
• Predictable timing in auditory perception.
• Neural mechanisms of timing.

## What music psychology reveals about the natural bounds of human temporal processing

Rhythm is an aspect of music that occurs on a medium temporal scale (hundreds of milliseconds to one or two seconds), longer than that of pitch (up to tens of milliseconds), but shorter than that of global musical form and structure (several seconds to minutes, e.g. phrases, sections, movements). Crucially, it is at the temporal scale of rhythm that a number of overt motor processes in humans tend to occur, such as the swing of the arms and legs during walking or the inhaling and exhaling of air during breathing. Dance, for example, is movement to the rhythm of music. In the Western music tradition, movements such as dance are typically synchronized to a periodic pulse, or beat. It is important to highlight that pulse and beat are not physical properties of the music itself, but are perceptual phenomena that arise from music through beat induction (BI). BI refers to our ability to extract a periodic pulse from music and is widely considered a cognitive skill, though its species-specificity and domain-specificity are topics of current debate (Honing, 2012). The neurophysiology underlying beat perception will later be discussed at length, but a brief review of music psychology research into perceptual aspects of rhythmic timing will first offer a number of practical observations from which to embark on this investigation.

### Timescales

Studies into sensorimotor synchronization (SMS) tend to employ simple movements such as tapping a finger as a readout of the perceived beat. These studies find that beat is generally perceived between 0.5 and 4 Hz, corresponding to time intervals of 250 ms to 2 s, a range beyond which precise coordination of motor movements becomes difficult (Repp, 2005, McAuley et al., 2006, Repp and Su, 2013). Even within this range, perception of time differs between shorter and longer time intervals. When asked to judge the duration of time intervals, there is a systematic tendency for human listeners to overestimate shorter time intervals (roughly 250–400 ms) and underestimate long ones (∼600 ms to 2 s). The transition point in between, measured by various researchers to lie between 400 and 600 ms, is termed the indifference interval and also corresponds to the rate at which people spontaneously tap (Fraisse et al., 1958; Fraisse, 1963; 1978, 1982; Clarke, 1999).

**Fig. 1:**
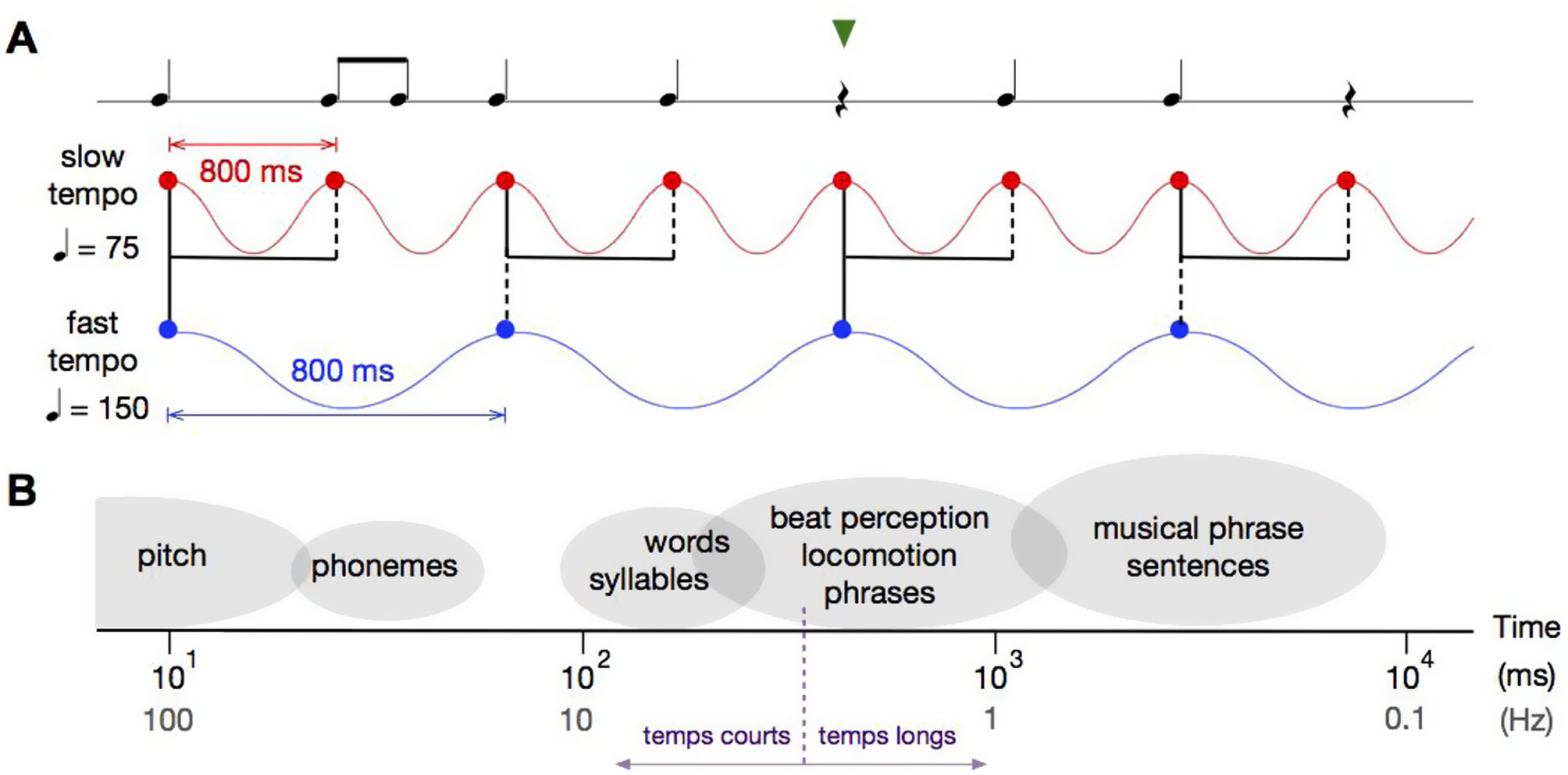
(A) The beat perceived depends on the tempo at which a musical rhythm is played. In this simple, recognizable example rhythm, notes represent sound events and those with a single stem are quarter notes, notes with the attached stem are eighth notes, and the remaining symbol is a quarter rest (silence). The basic unit of time here is the quarter note; a quarter rest is the same duration as a quarter note, and each eighth note is half the duration of the quarter note. Tempo is conventionally specified in beats per minute, so for the slow tempo (in red), there would be 75 quarter notes per minute, and each quarter note is therefore 800 ms in duration. The fast tempo (in blue) is twice the speed of the slow tempo. In both cases, the beat may be comfortably perceived at 800-ms intervals or 1.25 Hz (filled circles), but depending on the tempo this may coincide with different events in the music. The alternation of strong (solid lines) and weak beats (dotted lines) are illustrated for each tempo. Syncopation (green triangle), or when a beat is felt where there is silence, is very common in music. (B) This schematic illustrates the time scales over which common auditory events unfold. Time is on a log scale from small intervals (fast rates) to large intervals (slow rates), with values shown in milliseconds and in Hz. The indifference interval is marked in purple; shorter intervals are *temps courts*, longer intervals are *temps longs*.

In the context of rhythm perception, it is also the boundary between *temps courts* and *temps longs* (Clarke, 1999). When human subjects are asked to tap rhythmically, almost invariably they employ a 1:1 or 1:2 ratios to the time intervals between successive taps. A ratio of 1:2 refers to a tapping pattern of long and short intervals where the short intervals are precisely half the duration of the longer ones. This alludes to the theory that *temps longs* are intervals during which a listener is aware of the passage of time, whereas *temps courts* do not evoke a sense of time passage by themselves, but listeners are aware that a certain number of them grouped together make up a longer interval. Tapping ratios of 1:2 observed almost always span the indifference interval, with the longer interval belonging to *temps longs* and the shorter one to *temps courts* (see Fig. 1). A preference for time intervals with integer ratios also shapes the way rhythmic patterns are perceived (Jacoby and McDermott, 2017). Compared with intervals with noninteger ratios, intervals with integer ratios are more accurately reproduced by listeners (Essens and Povel, 1985) and show a distinct pattern of neural activity (Sakai et al. (1999); see also the later section entitled *Neurophysiology of beat perception*). Interestingly, while a preference for integer ratios spans different cultures, the specific ratios preferred by listeners is primarily determined by their music listening experience and is not strongly affected by musical expertise (Jacoby and McDermott, 2017).

### Beat – a perceptual accent

The timescales are one aspect of what determines where a musical beat might be felt, but not all sound events in music are equally likely to induce a beat percept. Certain events in music have been described as giving rise to perceptual accents, which, together with the temporal constraints described earlier, form the basis of where the beat is felt.

Perceptual accents may be felt at points that differ in loudness or in frequency relative to surrounding events. However, perceptual accents can also arise purely through temporal context. Essens and Povel (Povel and Essens, 1985) proposed a theoretical framework for metrical complexity based on empirical observations. They posit that (1) an isolated acoustic event will be perceived as accented, (2) the second of a set of two similar or identical acoustic events played in sequence will be perceived as accented, and (3) the first and last of three or greater similar events in a sequence will be perceptually accented. Based on the location of perceptual accents within a rhythm (which themselves may not be periodic), the period and phase of a periodic pulse can be determined.

### Not all beats are created equal, nor is there always an accent: subjective rhythmization

The basic temporal structure of a piece of music can be described by its meter, or its recurring pattern of *strong beats* and *weak beats.* Again, ‘strong’ and ‘weak’ in this context are perceptual notions, much akin to identical ticks of a clock being instinctively perceived as tick-tock-tick-tock (Bolton, 1894, van Noorden and Moelants, 1999, Brochard et al., 2003, Bååth, 2015). This tick-tock of a clock could be described as having a binary meter, or a beat pattern based on the number two (most commonly two or four beats in a bar) and have the beat pattern of strong-weak-strong-weak. Ternary meters, or bars based on the number three, have a pattern of strong-weak-weak-strong-weak-weak, the most common example being a waltz. Other more complex meters, for example based on 5 or 7 beats in a bar are also common in Western music, though binary and ternary meters are more often studied because they are generally more effective in inducing a clear beat percept. The preference or natural acceptance of binary meter could be due to a likeness of such meters to common rhythmic motor patterns such as breathing or walking.

### To summarize

Within the range of frequencies that a periodic pulse can typically be perceived, there is a further distinction between longer timescales across which the passage of time is noticeable, and shorter timescales of which several together are perceived to fit into a longer timescale. The boundary between the two is the indifference interval, which lies somewhere between 400 and 600 ms. This is where temporal perception is most accurate in humans (Fraisse, 1978), and incidentally also corresponds to a comfortable walking pace (Styns et al., 2007). Beats themselves arise as a result of the combination of perceptual accents and the constraint of a periodic pulse within the range of perceivable beat frequencies. A repeating pattern of strong and weak beats group together to form the musical meter of a piece, and some meters (binary and ternary) are more easily interpreted generally, perhaps due to their semblance to binary motor patterns or to the harmonic series on a fundamental frequency.

## The psychoacoustics of beat perception: sensorimotor synchronization

With beat and meter defined, we are now equipped to explore how we synchronize to beat. When we hear a beat in music, we almost instinctively want to move with it, and it has been shown that listeners often cannot maintain movements that are out of sync (Repp, 2002a). The synchronization of our movements to an external rhythm is known as sensorimotor synchronization (SMS). SMS has been studied extensively (see Repp, 2005 and Repp and Su, 2013 for reviews), and we highlight a few observations from SMS studies that may be of particular relevance to a discussion of the neurophysiological processes that underlie rhythm perception.

### When tapping along, we are usually early

Negative mean asynchrony (NMA) is a testament to the predictive nature of synchronizing a motor action with an expected stimulus. NMA refers to the observation that listeners, when asked to tap along with an isochronous pacing stimulus such as a metronome, tend to anticipate stimulus onsets with their taps by tens of milliseconds, rather than tapping with a distribution that is symmetric around sound onsets (sometimes early, sometimes late). Interestingly, listeners are often unaware of their own NMA, suggesting a general incongruence between objective and subjective synchrony. Musicians tend to show less NMA than nonmusicians, and the neurophysiological differences between the two groups may therefore shed some insight into the interaction between the sensory, motor, and cognitive processes involved. A final observation is that NMA decreases as the tempo of the pacing stimulus increases, which may allude to the tendency to overestimate short time intervals and underestimate longer ones described in the previous section on *Timescales*. For a more comprehensive review of NMA, see (Aschersleben, 2002).

### Beat period and phase may have distinct underlying representations

**Fig. 2:**
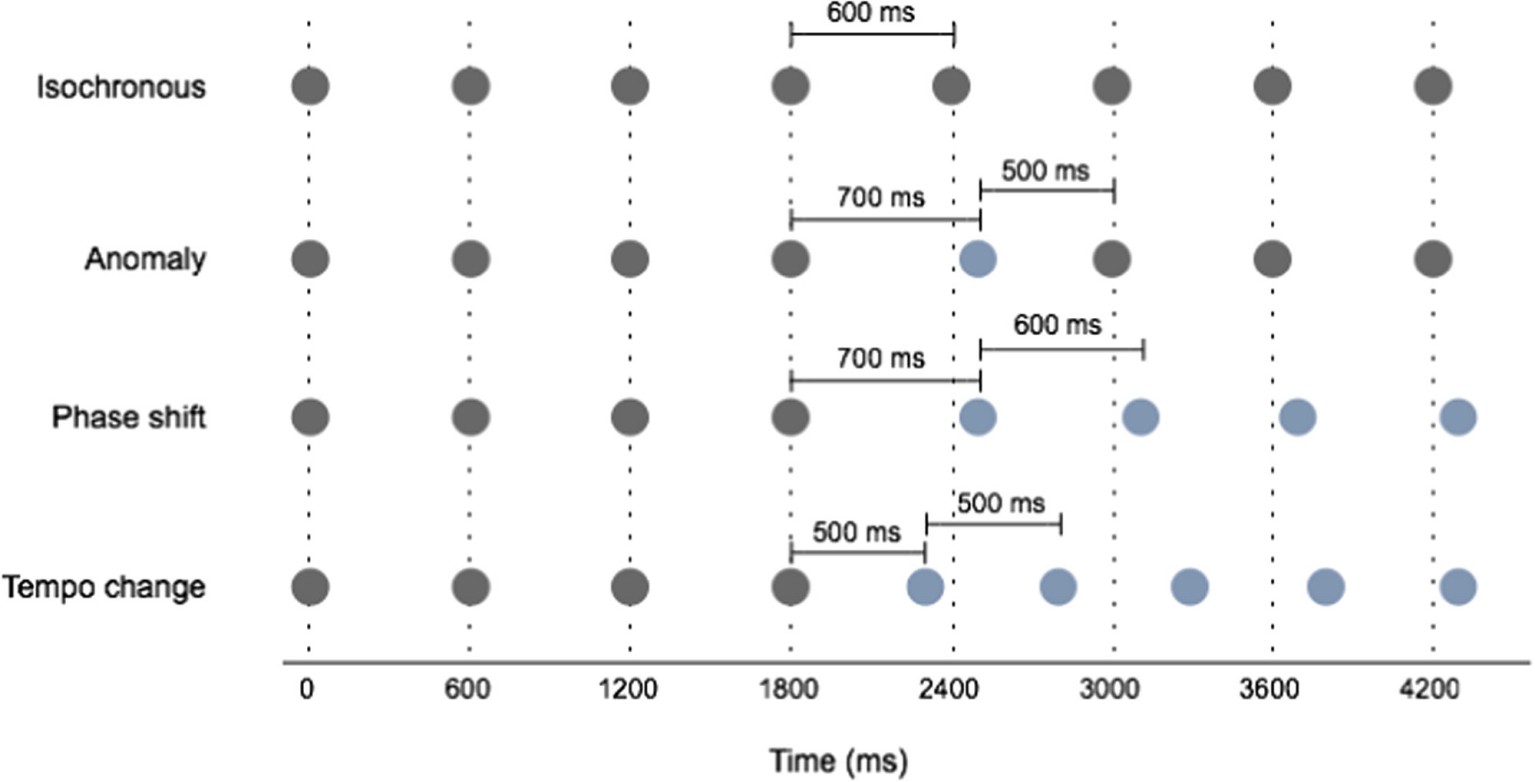
Illustration of a selection of perturbations used to study period and phase correction in sensorimotor synchronization. The *x*-axis represents time, and here a temporal grid representing 600-ms intervals is marked by the vertical dotted lines. Circles represent clicks to which a listener would align their taps, and blue circles mark sounds whose timing would be a departure from the isochronous condition where there is no perturbation. In the *Isochronous* condition (top), a click is played every 600 ms. In *Anomaly*, a single click in the sequence is manipulated such that the IOIs that flank it are too long and too short by 100 ms, allowing the remainder of the sequence to remain unchanged. In *Phase Shift*, a single IOI is lengthened by 100 ms but this time is not gained back, resulting in a phase shift of 100 ms that persists for all remaining clicks, even though their IOI remains 600 ms. In *Tempo Change*, the IOI changes abruptly from 600 ms to 500 ms. This would be perceived as a faster tempo and would require and adjustment in the period of taps.

A number of intriguing insights into SMS have also been uncovered through studies that systematically perturb the pacing stimulus, for example by introducing a phase offset, tempo change, or a sequence of distractors. Overwhelmingly, the evidence points to an interesting behavioral dissociation between phase correction and period correction (Repp, 2005). Phase correction in an isochronous sound sequence refers to a subtle adjustment of tapping so that it returns to synchrony following an unexpected inter-onset interval (IOI) that is abnormally short or long, which would result in an abrupt phase shift in the sequence that is either temporary or persistent (*Anomaly* and *Phase shift* in Fig. 2, respectively). Period correction refers to the adjustment of taps to a sudden tempo change, or an abruptly shorter or longer IOI (*Tempo change* in Fig. 2).

The phase correction mechanism appears to be automatic; the timing of the tap subsequent to the perturbation shifts according to whether the preceding IOI was shortened or lengthened, even when the phase offset is imperceptible to listeners (Repp, 2002b). Similarly, shifting a single tone in an isochronous stimulus such that it results in a shorter IOI on one side of it and a longer IOI on the other induces an involuntary shift in tap times after the perturbation, even when participants were told to ignore the perturbation. If a distractor sequence of isochronous tones is introduced, taps shift toward it, and interestingly this effect appears to be insensitive to the pitch difference between the tones of the pacing and distractor sequence. In this case, temporal coherence seems to be key: if a target and distractor tone are within 120 ms of each other, tapping behavior would suggest that they are treated as a joint referent. In contrast, period correction to a step change in tempo appears to require the change in tempo to be perceptible (Repp and Keller, 2004). Listeners’ ability to completely ignore a tempo change and continue tapping at the original tempo without showing any period correction is further evidence that period correction requires cognitive control, in contrast to phase correction, which in the same task proved impossible for participants to suppress. Under the looser constraint of self-paced movements, there does appear to be a natural tendency to synchronize movements to the period and phase of a musical beat (Peckel et al., 2014).

### How fast we can tap along depends on what we are tapping to

Depending on the nature of the task, different studies reports somewhat different ranges within which beat perception and SMS can occur. In truth, the context-dependent nature of SMS is in itself a reflection of the different sensory and biomechanical constraints. At the slow extreme, IOIs longer than 1.8 s result in inaccurate synchronization where taps begin to lag the pacing stimulus. At the fast extreme, finger tapping with an isochronous pacing stimulus can be done at a rate of up to 5–7 taps per second. However, if the task is 1:n synchronization, the IOIs in the pacing signal can be as short as 100–120 ms for trained musicians, which suggests that audiomotor processing can cope with these fast rates. This so-called subdivision benefit too depends on the exact subdivision required. 1:2, 1:3, 1:4, and 1:8 tapping can be done at lower IOIs than 1:5 or 1:7, with 1:6 and 1:9 tapping falling somewhere in between. This suggests a certain level of automaticity to subdivision by 2, 3, 4, and 8, while the cognitive demands of counting groups of 5 interferes with sensory processing. A similar effect is observed when listeners tap an isochronous beat in non-isochronous rhythmic patterns. Rhythmic patterns differ in their complexity, and while very complex rhythms are difficult to synchronize to (Povel and Essens, 1985), rhythmic patterns of medium complexity are what elicit the greatest desire from listeners to move (Witek et al., 2014). This may relate to beat salience, which has been shown to correlate with listeners’ desire to move (Madison et al., 2011). Rhythmic complexity and the strength of the beat percept also influence the precision of temporal judgments (Grube and Griffiths, 2009) and may be due to differences in neural representation of metrically simple, complex, and non-metrical sound patterns (Sakai et al., 1999, Vuust and Witek, 2014).

### To summarize

Perception appears to be based on a judgment of intervals, whereas action appears to be the result of a joint computation based on stimulus onsets and ongoing taps. Beat has both a period and a phase, and perturbation studies suggest that dissociable processes underlie adjustment of each. Specifically, phase correction appears to be automatic and involuntary, while period correction requires cognizance of a tempo change and can be suppressed at will. The temporal limits of synchronization ability are also context-dependent and are a result of biomechanical, sensory, and cognitive constraints. These factors are also at play in the context of more complicated rhythmic patterns and real music, where the temporal structure of the sound affects listeners’ ability and desire to synchronize.

## Neurophysiology of beat perception

A number of electrophysiological studies in humans have attempted to identify the neural correlate of the beat percept. Neural signatures of beat perception have been identified through direct and indirect means and involve distributed cortical and subcortical networks (Teki et al., 2012). Comparisons with studies in newborn humans (Winkler et al., 2009b) and nonhuman species would suggest that some aspects of rhythm perception may be innate to humans and to some nonhuman species, whereas other aspects may be unique to humans.

### Strong beats differ physiologically from weak beats

As described earlier, subjective rhythmization can generate a metrical percept of alternating strong and weak beats even in an isochronous sequence of identical sounds. This paradigm arguably would allow for the dissociation between the cognitive and the sensory aspects of beat perception in the context of identical isochronous sounds. Electroencephalography (EEG) studies that investigate the neural correlates of subjective accenting do so either directly or indirectly. Indirect methods involve the measurement of event-related potentials (ERPs) resulting from rare “deviant” (e.g. an omission or change in loudness) in a series of “standard” or expected sounds. Differences in the ERP to perturbations coinciding with strong and weak beats may therefore signify neurophysiological differences in processing that result from subjective accenting. Early components of the ERP are known as the mismatch negativity (MMN) and are considered to be pre-attentive in contrast to later components (300–600 ms post-stimulus onset), which are presumed to reflect cognitive mechanisms. Though subjective accenting is cognitive by definition, the setting of temporal expectations may influence the processing of forthcoming sounds in a predictive manner, and indeed both early and late ERP differences have been found between deviants at strong and weak beat positions (Brochard et al., 2003, Abecasis, 2005, Geiser et al., 2010, Schaefer et al., 2010, Bouwer et al., 2014; Honing et al., 2014).

The more direct approach compares sound-evoked responses at strong and weak positions in rhythmic sequences. Here too, strong beats evoke higher source current activity than weak beats in temporal and frontal areas, despite sounds being acoustically identical (Todd and Lee, 2015). Similarly, a target sound played over a background of pop music evokes stronger cortical and brainstem responses if it was presented on the beat, rather than shifted off the beat by ¼ of the inter-beat interval (Tierney and Kraus, 2013). All together, these event-based studies suggest that metrically strong positions are accompanied by larger source currents than metrically weak positions, and that these differences may be pre-attentive. It is worth noting, however, that by design these studies look at differences in predictions of not only “when” an auditory event is expected, but also “what” that auditory event should be (Teki and Kononowicz, 2016). Behavioral evidence suggests that these two types of predictions may have distinct neural substrates (Morillon et al., 2016, Rajendran and Teki, 2016), and it is therefore not yet possible to say whether pre-attentive responses are a result of temporal expectation alone or a combination of expectations of “what” and “when” (Arnal, 2012, Arnal and Giraud, 2012, Schwartze et al., 2013).

### Entrainment of oscillatory activity to musical beat

In addition to event-based descriptions of the beat percept, cortical oscillations have also been shown to reflect metrical structure. This is noteworthy because it suggests that neural oscillations, in addition to entraining to the rate of individual events in a rhythmic sequence, are also able to entrain to higher-level temporal regularities, but the precise mechanism behind this is still unknown. Modulation of auditory cortical activity in the beta band has been shown to track the clicks of a metronome, while gamma oscillations appear to encode anticipated stimulus timing as evidenced by a peak in gamma activity even in the absence of a click (Fujioka et al., 2009). Beta oscillations have also been demonstrated to encode beat and meter imagery (Iversen et al., 2009, Fujioka et al., 2015), and the dynamics of induced beta oscillatory activity both in humans (Teki, 2014) and in nonhuman primates (Bartolo et al., 2014, Bartolo and Merchant, 2015) (see the later section on *Beat processing in nonhuman species*), have been shown to vary according to the temporal regularity of sound sequences. In addition to beta, gamma band oscillations also appear to encode beat and meter (Snyder and Large, 2005, Zanto et al., 2006), and entrainment in the low-frequency delta-theta band (<8 Hz) has also been shown to correlate with years of musical training (Doelling and Poeppel, 2015). Low-frequency entrainment to the beat has also been observed in the bulk electroencephalogram signal (Nozaradan et al., 2011, Henry et al., 2014; see Zhou et al., 2016 for a guide on the interpretation of low-frequency components in the Fourier spectrum). A hierarchical organization of oscillatory activity in the auditory cortex is thought to facilitate temporal processing of auditory stimuli and coordinate activity between sensory and other brain areas (Lakatos, 2005). Cortical oscillations have furthermore been hypothesized to provide a mechanism for attentional selection and may be entrained by rhythmic auditory stimuli (Lakatos et al., 2008, Schroeder and Lakatos, 2009, Gomez-Ramirez et al., 2011, Lakatos et al., 2013).

### Brain areas involved in beat perception

In addition to the auditory cortex, musical rhythms have been shown to engage a number of distributed brain areas, including several that would traditionally be considered part of the brain’s motor system, and hence might not immediately be thought of as playing a key role in beat perception. These include the basal ganglia, supplementary motor area, striatum, cerebellum, sensorimotor cortex, and premotor cortex (Parsons, 2001, Grahn and Brett, 2007, Zatorre et al., 2007, Chen et al., 2008, Grahn, 2009, Teki et al., 2011). Engagement of motor-related areas appears to be automatic since it is observed consistently even when listeners are instructed not to make overt movements (Chen et al., 2008). Activation in auditory and motor areas furthermore correlates with individual differences in beat perception (Grahn and McAuley, 2009).

The activation of brain areas during beat perception depends on several factors including the duration of intervals (Lewis and Miall, 2003), temporal context (Teki et al., 2011), and task demands (Merchant et al., 2013). The core timing areas of the brain, specifically the striatum and the cerebellum (Ivry and Schlerf, 2008) are activated in perceptual timing depending on the temporal regularity of the sequences. For isochronous sequences, where a clear beat can be perceived, timing relies more on a network involving the striatum, while for jittered sequences, where the percept of a beat is negligible and intervals are encoded in an absolute manner, timing relies more on an olivocerebellar network (Teki et al., 2011, 2012). Examination of individuals who exhibit “beat deafness” (Phillips-Silver et al., 2011), a rare condition that is associated with poor motor synchronization and/or impoverished beat perception (Sowiński and Bella, 2013), provides further evidence that beat perception may recruit distinct circuits depending on the implicit/explicit timing aspect of the task (Bégel et al., 2017). The dissociation of striatal and cerebellar responses for beat-based versus duration-based sequences has recently been observed to hold not only for perception but also for working memory for single time intervals in sequences with different rhythmic structures (Teki and Griffiths, 2014, 2016).

Beat perception itself may be subcategorized into the processes of finding, continuing, and adjusting the beat, and the evidence points strongly toward the basal ganglia being involved in the continued representation of beat rather than its detection or adjustment (Chapin et al., 2010, Grahn and Rowe, 2013). In one fMRI study (Chapin et al., 2010), participants were played six cycles of each of a set of complex rhythm and were tasked with attending to the rhythm, holding it in memory over 12 s, then reproducing it by tapping. During the attending phase, the basal ganglia showed significant activation only if the auditory stimulus was attended to, and if sufficient cycles of the rhythm had passed for listeners to perceive the beat. The basal ganglia also remained active during the rehearsal period. Similarly, in another fMRI study (Grahn and Rowe, 2013) where beat and nonbeat rhythms were played consecutively, the preceding rhythm determined whether the beat in the subsequent rhythm, if any, was a continuation from the previous rhythm (beat continuation), was sped up or slowed down (beat adjustment), or needed to be found afresh (beat finding). Here, the basal ganglia were most active in beat continuation conditions and less active for beat adjustment conditions, with no apparent difference between the beat finding and the nonbeat (where no beat was present) conditions.

The superior temporal gyrus, premotor cortex, and ventrolateral prefrontal cortex show activity during beat detection and synchronization through tapping (Kung et al., 2013). When tapping to rhythmic sequences that contain syncopation (the absence of sound on a perceived beat, see Fig. 1), differences in activation of the premotor cortex, supplemental motor area, basal ganglia, and lateral cerebellum were observed, and these differences were present even when motor actions were not executed and the beat was simply imagined (Oullier et al., 2005). Syncopation is among the factors that determine how engaging listeners find a piece of music, and pleasant music appears to more effectively entrain neural responses in the caudate nucleus of the basal ganglia (Trost et al., 2014). Premotor and cerebellar areas are also more heavily recruited in response to subjectively more “beautiful” rhythms, and activity in the ventral premotor cortex (PMv) is enhanced by rhythms that are at a preferred tempo (Kornysheva et al., 2010). Repetitive transcranial magnetic stimulation (TMS) over the PMv changed people’s preferred tempo, suggesting that the PMv may be involved in beat rate preference (Kornysheva and Schubotz, 2011).

Findings from a number of functional imaging studies begin to allude back to some of the observations from early studies on temporal processing in the context of music. For example, beat induction is poorer for a slow (1500 ms) tempo compared to a faster one (600 ms), and activity in the basal ganglia, premotor and supplementary motor regions, and thalamus is correspondingly reduced (McAuley et al., 2012). This is consistent with accounts that the motor system is preferentially engaged in the measurement of sub-second time intervals (Lewis and Miall, 2003). Basal ganglia activity peaks around 500–600 ms (Riecker et al., 2003), which is comparable to the indifference interval and the rate of spontaneous tapping in humans (Repp and Su, 2013). The upper tempo limit to beat perception (∼200 ms) may be determined by the time constant for temporal integration (Loveless et al., 1996), which is comparable to the duration of auditory short term sensory memory, or “short auditory store” (Cowan, 1984). Recent work, however, suggests that temporal memory resources may not be fixed for a discrete number of items but flexibly distributed according to the number of intervals to be encoded in a sequence (Teki and Griffiths, 2014, Joseph et al., 2016).

### Model-based accounts of beat perception

A number of theoretical models have been proposed that capture neural and behavioral aspects of beat perception. Neural resonance theory is an influential computational model that consists of two sets of dynamic nonlinear oscillators, one that receives sensory input (an “auditory” layer) and one that receives input and projects back to the auditory layer (a “motor” layer). The interaction between these layers can be modeled as a dynamical system, and the results resemble both neurophysiological and behavioral aspects of beat perception (Large et al., 2015). Neural resonance theory is compatible with the dynamic attending theory, which postulates oscillatory fluctuations in attention (Large and Jones, 1999). The active sensing hypothesis (Schroeder et al., 2010) postulates similar interactions between the auditory and motor system (see (Henry and Herrmann, 2014) for a comparison of the two hypotheses). The “action simulation for auditory prediction” (ASAP) hypothesis goes a step further by suggesting that auditory perception is sharpened by the explicit simulation of periodic movement in motor planning regions of the brain (Patel and Iversen, 2014). The precise mechanism for beat induction remains unknown, though the entrainment of neural oscillations is a common thread between these competing hypotheses.

### Beat processing in nonhuman species

So far, the discussion has focused on findings from human studies. Beat perception studies in nonhuman species are numerous, but apart from notable exceptions such as a cockatoo (Patel et al., 2009) and a sea lion (Cook et al., 2013, Rouse et al., 2016), nonhuman species have shown little compelling evidence of being able to perceive and synchronize to the beat as precisely as humans (Geiser et al., 2014). Though chimpanzees appear to show some synchronization ability (Hattori et al., 2013), it appears to be weak and quite limited in tempo range compared to that of the human. This may be somewhat surprising given that humans are not the only species that relies on rhythmic sounds such as vocalizations and produces rhythmic movements. Indeed, some signatures of rhythm perception in humans have also been observed in macaques, such as interval duration-selective modulation of beta oscillations (Bartolo et al., 2014). In this and other related studies, the macaques were given a serial continuation task where they tapped along to a metronome and continued tapping at the same rate after the metronome stops. Though tap times tended to lag metronome clicks by 100–250 ms, these lags were shorter than the macaque’s reaction times, suggesting that there was a predictive element, though not strong enough to mimic the near-zero or negative lags in humans. Like in humans, beta oscillations in the basal ganglia (putamen) show preference for the continuation of a beat, and overall, similar timing circuits have been identified in both human and nonhuman primates, though macaques show better performance when synchronizing their movements to a visual rather than auditory metronome (Merchant et al., 2015). This is in contrast to a clear auditory bias in humans (Honing and Merchant, 2014). Larger responses in primary auditory cortex to tones at “strong beat” positions in a rhythmic sequence than to the same tones in a rhythmically irregular sequence have also been observed in macaques, in addition to enhanced deviance detection ability (Selezneva et al., 2013). However, this may be due to sensitivity to rhythmic grouping rather than to beat perception itself, since certain aspects of beat-specific neural activity observed in human adults and newborns are not observed in macaques (Honing et al., 2012). From the perspective of low-level auditory processing, firing rate adaptation as early as the midbrain results in higher average firing rates on the beat than off the beat; this may explain why some beat interpretations are more likely to be felt than others, and may also be a relevant precursor to the entrainment of cortical oscillations to beat (Rajendran et al., 2017).

### To summarize

Human imaging studies have provided glimpses into the complex and highly distributed neural dynamics that are set into motion by musical rhythms. A key conceptual advance is the finding that rhythmic sequences engage auditory and motor areas more strongly than arrhythmic sequences, even during passive listening and in the absence of movement. Another is that perceptually strong beats evoke stronger neural activity than weak beats, which suggests a close link between neural activity and perception. Underlying both are oscillatory processes that are capable of entraining to the beat, but are also coordinated across sensory, frontal, parietal, and motor-related areas both cortically and subcortically. Some of these neural dynamics have been observed in nonhuman primates, and it therefore remains an open question to what extent humans are unique in their ability to perceive musical beat, and what differences in connectivity and neural response dynamics give rise to humans’ seemingly superior ability to spontaneously synchronize movements to music.

## Predictable timing in auditory perception

As alluded to in the introduction, an appreciation of music is only one of many consequences of our ability to perceive rhythmic patterns. We now begin to shift our focus away from music to explore rhythm perception in a more general context. Intrinsic to the perception of a musical beat is the prediction of when the next beat will occur, and the perceptual advantages afforded by our general ability to form temporal predictions will be the subject of this section.

### Temporal predictability in pattern detection

Humans show a remarkable ability to detect repeating patterns that are quite complex in their acoustic content (Agus et al., 2010, McDermott and Simoncelli, 2011, Kumar et al., 2014, Barascud et al., 2016). To do so is an impressive feat; the brain must be able to hold arbitrary sounds of arbitrary length and complexity in memory over timescales that can range from milliseconds up to tens of seconds (Kaernbach, 2004). It is therefore relevant that a feature of repeating sounds in nature is that they tend to be rhythmic and indicative of animate sound sources. Rhythm detection may therefore be an advantageous sensory capability, and it has been shown that rhythmic presentation of repeating sounds facilitates detection of complex acoustic patterns (Rajendran et al., 2016) and decreases detection thresholds (Lawrance et al., 2014).

The entrainment of oscillatory activity in the brain, mentioned earlier in the context of beat perception (see *Entrainment of oscillatory activity to musical beat*), provides a likely explanation for these results too. Rhythmic input is widely thought to entrain attentional resources (Lakatos et al., 2008, Bolger et al., 2013, Calderone et al., 2014) such that neuronal excitability is highest when the next stimulus is predicted to occur (Lakatos et al., 2009, Besle et al., 2011). Low-frequency entrainment of oscillations may therefore serve as a mechanism for sensory selection (Schroeder and Lakatos, 2009) and improve the quality of sensory information received (Rohenkohl et al., 2014). It is worth noting that the rhythmic form of temporal expectation is just one of several forms of temporal expectation, each resulting in subtle differences in perception that may arise from differences in the underlying neural substrates (Nobre et al., 2007, Breska and Deouell, 2017). For example, an enhancement of perceptual sensitivity has been demonstrated in both periodic and non periodic sequences that are temporally predictable, but motor facilitation through faster response latencies were only observed in the periodic condition (Morillon et al., 2016, Rajendran and Teki, 2016).

However, it is also important to note that there is an as yet unresolved tension, or apparent conflict, in the physiological literature regarding the nature of the neural responses involved in the processing of periodic or rhythmic stimuli. The aforementioned studies posit that entrainment due to temporal expectation and attention would result in periods of heightened sensitivity in phase with the rhythm, which would be expected to lead to enhanced, stronger responses. This is in contrast to well documented phenomena such as “repetition suppression” in auditory-evoked responses measured through EEG (Costa-Faidella et al., 2011) and “stimulus-specific adaptation (SSA)” observed in neural responses recorded extracellularly in auditory cortex and non-lemniscal parts of the inferior colliculus and thalamus (Malmierca, 2014, Khouri and Nelken, 2015, Nieto-Diego and Malmierca, 2016), which find that responses to simple periodic stimuli are reduced or suppressed, rather than enhanced. How can isochronous stimuli on the one hand produce entrainment that is suggestive of periodically heightened sensitivity but at the same time elicit reduced response amplitudes as evidenced by SSA or repetition suppression? The simple answer is that we do not yet know. The methodologies of studies of entrainment versus SSA are too different to allow direct comparisons. Entrainment studies typically use EEG or LFP measures, the amplitude of which depends at least as much on the degree of synchronization of neural activity as on net response amplitudes of individual neurons. Additionally, they are often carried out on awake human volunteers or animal subjects who may be attending to the rhythmic sounds, while SSA studies typically use anesthetized preparations to measure extracellular response amplitudes that are essentially independent of neural synchrony. Consequently, while the take-home messages from studies of entrainment and of SSA at present appear somewhat contradictory, how they may be reconciled will need to be addressed in future studies using unified methodologies.

### Temporal predictability in auditory scene analysis

Another practical advantage of forming predictions based on temporal patterns is that it allows us to parse a complex auditory scene into distinct perceptual objects (Winkler et al., 2009a). In addition to temporal coherence of sound features (Turgeon et al., 2002, 2005; Shamma et al., 2011), the predictability of features such as location, pitch, loudness, and timbre play a pivotal role in auditory scene analysis (Bendixen, 2014). The segregation of a set of sounds from another set of sounds is known as auditory stream segregation and has often been probed experimentally using an alternating tone paradigm of A-B-A, where A and B tones are typically different frequencies of a certain frequency separation (Bregman and Campbell, 1971, van Noorden, 1975). Temporal regularity within these paradigms influences whether these sequences are perceived as integrated (A-B-A-B-A) or whether they segregate into two perceptually distinct streams (A---A---A and --B---B) (Bendixen et al., 2010, Andreou et al., 2011, Rajendran et al., 2013). Together with attentional effects, predictive coding based on temporal and other feature regularities may account for the stability of auditory objects (Denham and Winkler, 2006, Pressnitzer and Hupe, 2006, Chait et al., 2007, Winkler et al., 2012).

Current theories suggest that the formation of auditory objects may rely on both basic sensory neural mechanisms (Fishman et al., 2012) and attention-driven oscillatory mechanisms (Lakatos et al., 2008, Schroeder and Lakatos, 2009). Though the question of how a perceptual object is formed from potentially noisy and conflicting information is still an open one, the final representation of an auditory object is remarkably distinct and robust, even if it overlaps spectrotemporally with the unattended background (Ding and Simon, 2012, 2013). A key question here, which may also be relevant to how beat and meter emerge from music, is whether and how different oscillatory populations of neurons entrain to different time-varying sound features, and how their relative contributions are weighted and integrated to form a coherent percept of a single speaker in a noisy room.

### Rhythms in speech perception

Speech is perhaps the most pervasive and critical context in which we rely on our ability to derive meaning from complex temporal patterns. The intelligibility of speech has been shown to correlate with the entrainment of oscillatory neural responses to the speech envelope (Ahissar et al., 2001, Peelle and Davis, 2012), particularly in the 4–8-Hz range (Luo and Poeppel, 2007). This range corresponds to the syllable rate of speech production (Greenberg et al., 1999) and dominates the temporal modulations in the speech envelope (Chi et al., 1999, Chandrasekaran et al., 2009, Elliott and Theunissen, 2009). The syllabic rate is nearly an order of magnitude slower than fine structure elements such as formants (30–50 Hz), and a few-fold faster than intonation contours that are typical of phrasal units (1–2 Hz). Content at all of these timescales are parsed concurrently to extract meaning.

Speech, like music, is built hierarchically from elements that span short to long timescales. A recent survey of temporal modulations in speech and music reveals a consistent peak in temporal modulations around 5 Hz for speech across nine languages, and around 2 Hz for music across several (Western) musical genres (Ding et al., 2017). It is worth emphasizing, however, that the temporal structure present in speech is not periodic like it is in music (Nolan and Jeon, 2014). The temporal modulations in speech are constrained by the motor system though, specifically by the biomechanics of the articulators, and this results in clear temporal structure in both the auditory and visual components to speech (Chandrasekaran et al., 2009). There is strong evidence that speech contains sufficient temporal structure to robustly entrain oscillatory neural activity (Giraud and Poeppel, 2012), and that this entrainment serves to maximize processing efficiency of future inputs by ensuring that intervals of high neuronal excitability coincide with when critical information is expected to arrive (Peelle and Davis, 2012, Ding et al., 2017).

Interestingly, temporal manipulations to speech more drastically impair intelligibility (Adank and Janse, 2009) than extreme spectral manipulations do (Shannon et al., 1995). Model-based accounts (Ghitza, 2011, Giraud and Poeppel, 2012) suggest that phase-locking and nested theta-gamma oscillations could explain why an extremely impoverished speech signal can be understood if the syllabic rhythm is preserved (Ghitza and Greenberg, 2009). The “asymmetric sampling in time” (AST) hypothesis suggests that the two cerebral hemispheres sample an auditory signal at different rates; the left auditory areas extract information from 20 to 40-ms temporal integration windows, while auditory areas in the right hemisphere sample using 150–250-ms temporal integration windows (Poeppel, 2003). A related hypothesis suggests that the left hemisphere has better temporal resolution and the right hemisphere has better spectral resolution, and that this functional organization reflects an optimization of processing for speech and music, respectively (Zatorre et al., 2002). Both of these ideas are consistent with the observation that the left hemisphere dominates during speech processing while the right hemisphere dominates during music processing (Tervaniemi and Hugdahl, 2003). The parallels drawn here between music and speech deal strictly with timing and do not suggest that music has any meaning that is analogous to the semantic meaning of speech (Lerdahl and Jackendoff, 1983). However, given these parallels, it is possible that music and speech co-evolved (Fitch, 2000, Hauser and McDermott, 2003, Fitch, 2006, Patel, 2007) and are built on overlapping circuit mechanisms for auditory working memory (Hickok et al., 2003, Joseph et al., 2016) and timing (Patel, 2011).

### To summarize

The temporal predictability that results from rhythmic stimulation helps us detect patterns, parse an auditory scene into distinct auditory objects, and understand speech. Entrainment of neural oscillations, which by virtue of aligning to temporal modulations in a rhythmic acoustic signal generates predictions about future input, is thought to underlie all of these abilities. The acoustic stimuli used in these studies range from extremely simple alternating tone paradigms, to the parsing of two people speaking simultaneously. Much remains to be understood regarding what periodically or quasi-periodically repeating features in a spectrotemporally complex sound entrain oscillations, and how such oscillations are ultimately integrated to form distinct auditory objects or extract meaning. Knowing these answers would likely shed light on the mechanism and functional role of oscillatory entrainment to musical rhythms.

## Neural mechanisms of timing

Music, speech, and the parsing of complex auditory scenes all rely on an ability to detect temporal regularities in order to form the temporal predictions that drive how sounds in the future are perceived. This requires some form of timekeeping in the brain. The timing field is vast and is a likely reflection of the complexity of the neural circuits that are able to track time (Teki, 2016). Those findings most relevant to our discussion are reviewed here.

### Dedicated timekeeping circuits?

The neuronal mechanisms underlying timing have been a subject of investigation for several decades. Braitenberg (1967) proposed the cerebellum as a biological clock in the millisecond range. Since then, the concept of a central clock or internal timekeeper has dominated timing research. Early work highlighted the unique synaptic circuitry of the cerebellum and the inferior olive as being capable of generating precise timing signals. Specifically, inferior olive neurons, which provide climbing fiber input to the Purkinje cells in the cerebellum, possess unique voltage-gated conductances that exhibit rhythmic sub-threshold membrane potential oscillations (5–15 Hz) as well as electrical gap-junctions that synchronize membrane potential oscillations across cells into distinct neuronal clusters that show temporally coherent activity (Llinas et al., 1974, Llinas and Yarom, 1981). The deep cerebellar nuclei like the dentate nucleus modulate the electrical activity of olivary neurons and decouple them into dynamic cell assemblies. Furthermore, these deep cerebellar nuclei are inhibited by the Purkinje cells, completing a feed-forward inhibitory loop. These neurophysiological properties provide the olivocerebellar network with the capacity to generate accurate absolute timing signals for motor and perceptual timing (Welsh et al., 1995, Yarom and Cohen, 2002, Jacobson et al., 2008, Mathy et al., 2009). The use of timing signals from the olivocerebellar network has been demonstrated across several timing paradigms in human studies as well (Xu, 2006, Teki et al., 2011, Wu et al., 2011, Lusk et al., 2016).

Motivated by neuropsychological evidence from Parkinson’s patients who showed perceptual timing deficits, parallel work focused on the basal ganglia as a core timing network in the brain (Artieda et al., 1992). Matell and Meck (2004) proposed an oscillatory timing model: medium spiny neurons in the dorsal striatum act as coincidence detectors of oscillatory cortical activity (5–15 Hz; Miall, 1989). The cortical oscillations are proposed to be synchronized at interval onset by phasic dopamine release from the ventral tegmental area, while dopaminergic input from the substantia nigra modulates the activity of the dorsal striatum (Buhusi and Meck, 2005). Cortico-striatal synapses are strengthened or weakened over experience through long-term potentiation and depression, and after repeated stimulus presentation, medium spiny neurons learn to encode the duration of reinforced time intervals (Gu et al., 2011). Several studies point to the importance of the dopaminergic basal ganglia network in mediating accurate timing signals (Jin et al., 2009, Bartolo et al., 2014, Gershman et al., 2014, Chiba et al., 2015, Gouvêa et al., 2015, Mello et al., 2015, Soares et al., 2016).

Cortical networks such as primary visual, auditory, parietal and frontal cortices have also been implicated in sensory timing functions (e.g. Leon and Shadlen, 2003, Bueti et al., 2008, Bueti and Macaluso, 2010; Bueti, 2011; Schneider and Ghose, 2012, Hayashi et al., 2015, Jazayeri and Shadlen, 2015, Namboodiri et al., 2015, Bakhurin et al., 2016, Shuler, 2016). However, it is not completely understood what aspects of timing are respectively mediated by each of these networks, nor are the dynamics of temporal processing across sensory and higher order cortical networks completely clear (see Rao et al., 2001). The likely hypothesis is that early stage sensory cortices process the stimulus-related features while parietal and frontal cortices are engaged by task demands like memory and attention (Finnerty et al., 2015). More recently, the hippocampus (CA1) has been shown to have ‘time cells’ that display increased firing rates in relation to elapsing durations, independent of space and distance (MacDonald et al., 2011). The prevailing view suggests the existence of ‘time cells’ in the striatum, cerebellum and the hippocampus whose output is integrated to obtain a common percept of time (Lusk et al., 2016).

### Is the passage of time implicit in neural responses?

An alternative hypothesis to the one positing that time is kept by dedicated sensorimotor circuits is one that suggests that timing is an intrinsic computation that emerges from network-wide neural dynamics (Goel and Buonomano, 2014). Hardy and Buonomano (2016) have recently reviewed a number of plausible neurocomputational models of timing. Here, we briefly summarize the primary models and their principles of operation.

One of the simplest network models of timing is based on ‘synfire chains’ where groups of neurons are connected in a feed-forward fashion such that each neuronal population is activated at a different instant in time (Haß et al., 2008). Synfire chains represent neurobiologically plausible mechanism for interval timing but are limited because of their pure feed-forward architecture and absence of recurrent connections. Positive feedback models, on the other hand, use recurrent excitatory connections and are compatible with experimental findings on cortical representation of time (e.g. Namboodiri et al., 2015). The limitation of these models, however, is that it is not known whether these can be generalized to sequences of intervals. Finally, state-dependent networks of timing and temporal processing are based on the hypothesis that sensory events interact with current states of recurrent networks to form a sequence of network states that encode each event in the context of recent stimulus history (Karmarkar and Buonomano, 2007). Several studies have demonstrated that cortical networks can be trained to represent time intervals in the hundreds of millisecond range where timing is proposed to emerge from network-wide and pathway-specific changes in evoked neural dynamics (e.g. Goel and Buonomano, 2016).

### To summarize

Although the notion of population clocks is gaining traction (Hardy and Buonomano, 2016), there is no compelling biologically plausible model that generalizes these results from studies based on computation of single time intervals to complex sequences such as those observed in music. Natural motor commands as well as sound sequences like speech and music consist of dynamically varying time intervals with different temporal structures. Several of the circuits reviewed above are specialized for processing time on the scale of tens of milliseconds to a few seconds, but it is not yet clear which of these mechanisms apply in the context of beat perception as this has not directly been tested. Integrating basic mechanisms of sound processing observed along the auditory pathways with models of timing may provide some novel insights into the examination of pattern timing.

Timing functions are distributed across the brain and are expressed in subcortical motor structures like the basal ganglia and the cerebellum, sensory and motor cortices, as well as higher order areas like the parietal and frontal cortical networks. While the timing computations performed by of each these individual brain regions is not fully understood, it is evident that particular areas are specialized for mediating specific timing functions. Future research may benefit from dissecting the precise role within each brain area and as a part of a distributed timing network.

## Conclusions and outlook

A lot of ground has been covered in this review, largely because the work comprising each section draws from a different field (or several different fields) of research that have so far shown little overlap with the others. This is despite these topics sharing common themes that unite them. For example, the timescales that are relevant in music are also relevant in other contexts such as in the production and perception of movement (walking, running, breathing) and speech, and in the parsing of complex acoustic scenes (see Fig. 1B). Furthermore, the entrainment of neural oscillations through sensorimotor loops may be a central mechanism governing perception and action in all of these contexts. By presenting an overview of these diverse topics that likely rely on similar temporal encoding mechanisms, we hope that this review will provide an insightful point of departure for future investigations into auditory temporal sequence processing.

We conclude by leaving the reader with an open question that we believe will be pivotal to advancing our understanding of temporal sequence processing, namely a mechanistic understanding of the entrainment of neural oscillations. While a large body of work points to the importance of neural oscillations (the studies mentioned in the second half of this review only scratch the surface), this topic is nevertheless not without controversy with many questions that remain unresolved, starting with the functional role that oscillations in different frequency bands play in information coding and retrieval. A number of theories have been proposed that describe functional aspects of oscillatory dynamics, including communication between neuronal groups through coherence of oscillations (Fries, 2015), the prioritization of sensory streams through pulsed inhibition via alpha oscillations (Haegens et al., 2011, Mathewson et al., 2011, Jensen et al., 2014, Strauß et al., 2014), the retrieval of memories through spiking that is phase-locked to theta oscillations (Hsieh and Ranganath, 2014), and the active sensing of sound through rhythmic temporal priors provided by the motor system (Morillon et al., 2015). Of particular relevance are the behavioral (Morillon et al., 2016) and neuronal dissociations (Breska and Deouell, 2017) that have been observed between auditory sequences that are periodic versus temporally predictable but not periodic, suggesting that the underlying neural dynamics manifest differently according to the nature of the temporal predictions being maintained. Further work is required to develop a unified understanding of the function served by the entrainment of neural oscillations.

A second question relates to the dynamics of entrainment itself, specifically how entrainment arises and unfolds, how it extracts higher-order temporal regularities in a rhythmic sequence, how it behaves in response to new sensory input, and how possibly different and simultaneous processes interact to guide what we perceive. Much of our current knowledge about the role of neural oscillations and entrainment in the perception of temporally structured stimuli is based on the interpretation of data obtained with non-invasive techniques (EEG, MEG, fMRI), which lack the fine resolution required to provide insights into these phenomena at the level of individual neurons and neural networks. Deeper insights will need data obtained at higher spatial resolution, as is typically obtained from invasive recordings in experimental animals, but the use of richly structured auditory stimuli such as music in animal electrophysiology experiments remains a highly unusual thing to do (see Rajendran et al., 2017 for a first step in this direction). However, we would suggest that employing music, in addition to traditional paradigms, may be especially fruitful since much is known already about our perception of music (see the first two sections of this review), and because it is a finely controllable stimulus paradigm within which nested periodicities across different sound features (frequency, loudness, duration) can be simultaneously present and tuned. Furthermore, we suggest that, since nonhuman model organisms do show some capacity to perceive and discriminate rhythms (see the section on *Beat Processing in Nonhuman Species*), and since recognizing rhythmic patterns in environmental sounds such as footsteps or vocalizations is of great importance to a wide range of organisms, complementary studies in nonhuman species should begin to fill in the gaps in our knowledge that non-invasive psychoacoustic and physiological studies on humans alone cannot answer.
